# Affective Balance, Team Prosocial Efficacy and Team Trust: A Multilevel Analysis of Prosocial Behavior in Small Groups

**DOI:** 10.1371/journal.pone.0136874

**Published:** 2015-08-28

**Authors:** Esther Cuadrado, Carmen Tabernero

**Affiliations:** Department of Psychology, Faculty of Educational Sciences, University of Cordoba, Córdoba, Spain; Tianjin University of Technology, CHINA

## Abstract

Little research has focused on how individual- and team-level characteristics jointly influence, via interaction, how prosocially individuals behave in teams and few studies have considered the potential influence of team context on prosocial behavior. Using a multilevel perspective, we examined the relationships between individual (affective balance) and group (team prosocial efficacy and team trust) level variables and prosocial behavior towards team members. The participants were 123 students nested in 45 small teams. A series of multilevel random models was estimated using hierarchical linear and nonlinear modeling. Individuals were more likely to behave prosocially towards in-group members when they were feeling good. Furthermore, the relationship between positive affective balance and prosocial behavior was stronger in teams with higher team prosocial efficacy levels as well as in teams with higher team trust levels. Finally, the relevance of team trust had a stronger influence on behavior than team prosocial efficacy.

## Introduction

Promotion of prosocial behavior—defined as an extensive category of intentional behaviors intended to benefit others [1]—encourages the development of networks that facilitate coexistence and wellbeing in healthier social and environmental contexts. This implies that social groups and teams would benefit from promoting prosocial behavior among members and hence that understanding the motivational determinants of prosocial intragroup interactions is important. Further, it would be pertinent, as well, to consider not only the individual-level variables that may influence prosocial behavior towards in-group members, but also the team-level variables that facilitate prosocial behavior towards fellow team members. Good team function may (a) promote prosocial behavior—just as it enhances team performance [2]—and (b) moderate the association between certain individual variables and prosocial behavior—just as it moderates the association between individual variables and performance [2]. In this study we analyzed the role of both individual- and group-level variables in the promotion of prosocial behavior in the context of small groups.

One of the variables traditionally related to prosocial behavior is affective state. Several studies have argued that positive affect promotes prosocial behavior [3, 4] whilst negative affect reduces prosocial behavior [5, 6]. We expected to confirm these associations, but we wondered whether other variables could influence the association between affective state and prosocial behavior, either reinforcing or attenuating it. We also know that a group or team is powerful enough to influence individuals and hence we wondered if the association at individual level between positive affect and prosocial behavior might be influenced by team variables. We are not aware of any studies previous attempts to investigate this potential moderation. Nevertheless, in order to promote prosocial behavior within groups, we consider the analyses of the potential influence of group level variables on the relationship between affective state and prosocial behavior an issue of theoretical and practical interest.

It has been shown that group efficacy [7–9] and trust [10–12] are associated with prosocial behavior. Again, we expected to confirm these associations. In an attempt to determine the strength of the influence of certain team variables on the association between affective state and prosocial behavior, and following on from work by Tasa et al. [13] we also suggested that team efficacy and team trust might create a positive atmosphere within a group that might in turn strengthen the association between affective state and prosocial behavior. We also followed Bandura [7] in predicting that team trust might be a stronger moderator of this association than team efficacy.

### Individual- and Team-level Predictors of Prosocial Behavior

Affective states have been found to influence thinking and interpersonal relationships [14]. There is currently considerable interest in how affective states influence prosocial behavior [15]. Several studies have shown how [3, 4] and explained why [16] positive affect enhances prosocial behavior. As Isen [14] has pointed out several studies have shown that positive feelings promote a wide range of helpful and generous behaviors. Moreover, it has been shown that negative affect increases antisocial behavior [5, 6] and decreases prosocial behaviors [17]. This body of research suggests that at individual level affective balance, i.e. a globally positive affective state [18] seems to be a reliable predictor of prosocial behavior (H1).

Certain group-level variables also predict prosocial behavior. Bandura’s efficacy theory [7] has provided empirical evidence about the impact of self- and group efficacy on behavior. At individual level, empathic self-efficacy has been shown to predict prosocial behavior [8]; and at group level, group efficacy has been shown to predict cooperation and communication [9]. We therefore propose that team prosocial efficacy—a team’s shared belief in its conjoint ability to act prosocially—would be a prerequisite for individuals to behave prosocially with their group members. By applying theself- and collective-efficacy theory [7], in teams with low confidence in the group’s ability to be prosocial—low prosocial team-efficacy—it will be unlikely that individuals will engage in such prosocial behavior with their group members. Thus, we hypothesized that individuals in teams with high team prosocial efficacy would behave more prosocially towards their group members (H2).

Trust, is a psychological state that “represents confidence in the strength of a partner’s commitment” [19] (p.339), and is defined as an expectation about others’ benevolent motives [20]. Trust has several benefits for teams and their members [21]. Costa [22] argued that trust has an important role in the functioning of teams and organizations. Trust can be defined as a social orientation towards other people and towards the group members as a whole. Balliet and Van Lange [23] argued that Trust—as an important source of social capital within social systems such as teams—engenders spontaneous sociability, operationalized in terms of the numerous forms of cooperative, altruistic, and extra-role behavior in which members of a group engage. The trust team members place in one another or in the team as a unit may influence how prosocially they behave towards members of their team. Most research on this issue has provided evidence that trusting people are especially willing to engage in prosocial behavior [10–12, 24], either because it was associated with an expectation of being rewarding with a sense of belonging [25,26] or because they expected reciprocity [27]. And Yamagishi [20] noticed that when it is difficult to trust is when trust is most needed to create a cooperative relationship. Moreover, Wang et al. [28] have shown that “the cooperator’s frequency (…) does not monotonously depend on the size of neighborhood, which assumes an important role in the emergence of cooperation” (p. 727). In fact, when considering networks, medium-sized groups may be the optimal size for maintaining intra-group cooperation; other factors also interact with group size to influence the cooperation ratio [28]. In this context it is also relevant that Sato [29] has shown that the effects of trust decrease as group size increases. We therefore predicted that in small teams with high team trust—a team’s shared beliefs in the confidence of the interactions with the team members—team members would be more willing to behave prosocially towards one another (H3).

In line with the literature, we predicted that:

Hypothesis 1: individuals with a globally positive emotional state would be more inclined to behave prosocially towards group members
Hypothesis 2: members of a team with high team prosocial efficacy would be more likely to behave prosocially towards one another
Hypothesis 3: members of teams with high team trust would be more likely to behave prosocially towards one another.

### Cross-level Relationships

Regarding the cross-level moderation, very little research has focused on how individual- and team-level characteristics jointly influence, via interaction, how prosocially an individual behaves in teams [30, 19]. As Lopes-Costa et al. [31] have pointed out “despite multilevel research being advocated by many researchers, it is not yet a very common practice;” (p. 8) and few studies have considered the potential influence of team context on prosocial behavior [32]. In this sense, an important aspect of our multilevel study pertained to analyzing the cross-level influences between individual- and team-level variables.

Tasa et al. [13] demonstrated that group efficacy exerted cross-level effects on the personality-behavior association by moderating its interaction. Similarly, efficacy beliefs have been shown to influence whether individuals are optimistic or pessimistic about something and their emotional responses [7]. Individuals with low personal efficacy with respect to task and individuals who felt that their group had low efficacy felt bad thus activating a negative emotional state [33]—and suffered a drop in positive affect [34]. We therefore hypothesized that, at team level, team efficacy interacts with and influences the association between individuals’ emotional state and individual prosocial behavior. This study addressed a gap in previous literature, by assessing how team-level variables influence an individual’s behavior—especially prosocial behavior—towards in-group members [13, 32, 35].

According to the trait-based interactionist model [36], the personality traits of individuals are expressed behaviorally (activated) in responses to trait-relevant situational cues. Tasa et al. [13] proposed that the positive atmosphere created by team efficacy might activate and increase the probability that an individual with a globally positive affective state will behave in a prosocial way. On the basis of the trait-based interactionist model [36] and the Tasa et al. [13] explanation, we posited that higher team prosocial efficacy would enhance the positive association between affective balance and prosocial behavior by providing situational cues that promote the activation of a positive global emotional which is then expressed behaviorally as prosocial behavior. We posited that at team level, team prosocial efficacy would strengthen the relationship between an individual’s positive global emotional state and his or her prosocial behavior. Team efficacy was expected to moderate the association between affective balance and prosocial behavior, such that team efficacy would strengthen the association between positive affective states and prosocial behavior (H4).

It has been shown that trust influences the emotional responses of individuals: trusting people tended to feel less pain when excluded from a group than less trusting people [37]. We inferred from this that individuals in high-trust teams would have a more positive emotional state than individuals in low-trust teams. We suggested that just as team efficacy creates a positive atmosphere in the group that may increase the strength of the association between affective balance and prosocial behavior [13], high team trust might create a similarly positive atmosphere, with a similarly positive effect on the positive affect-prosocial behavior association. In other words, the positive atmosphere created by trust would provide trait-relevant situational cues, which would activate [36] the inclination of individual’s in a positive affective state to act prosocially (H5).

Trust and perceived efficacy are related concepts [38]. Trust can enhance perceived efficacy [38]. Furthermore, Bandura [7] has demonstrated that the conjoint influence of collective perceived efficacy and trust predicts individual behavior. Bandura showed that group efficacy might be insufficient to induce individuals to participate actively in a political movement; without minimum level of trust, individuals in groups with high group efficacy are unlikely to engage in group political activity [7]. We assumed that prosocial behavior in a team context would similarly require a minimum level of team trust. It is plausible that if team trust is low—creating a negative atmosphere—then even if the team perceive a high conjoint capability for prosocial behavior as a unit (i.e. high team prosocial efficacy) the lack of trust will reduce (a) the probability that team members will behave prosocially towards one another and (b) the positive effect that globally positive emotional state has on prosocial behavior. Thus, team trust levels may have an important influence on the interaction of positive affect, team efficacy and prosocial behavior described above; team trust may enhance the positive influence of team prosocial efficacy on the relationship between affective balance and prosocial behavior, by amplifying the slope in trusting teams (H6).

In line with existing evidence we predicted that:

Hypothesis 4: Team efficacy would moderate the relationship between affective balance and prosocial behavior, such that individuals with a globally positive emotional state would be even more inclined to behave prosocially towards team members if the team has high team prosocial efficacy
Hypothesis 5: Team trust would moderate the relationship between affective balance and prosocial behavior, such that individuals with a globally positive emotional state would be more inclined to behave prosocially towards team members if the team had high team trust
Hypothesis 6: Team trust would moderate the cross-interaction between team prosocial efficacy, affective balance and prosocial behavior which is the subject of H4, such that individuals with a globally positive emotional state who belong to a team with high team prosocial efficacy would be more inclined to behave prosocially towards team members if the team had high trust.

**Fig 1:**
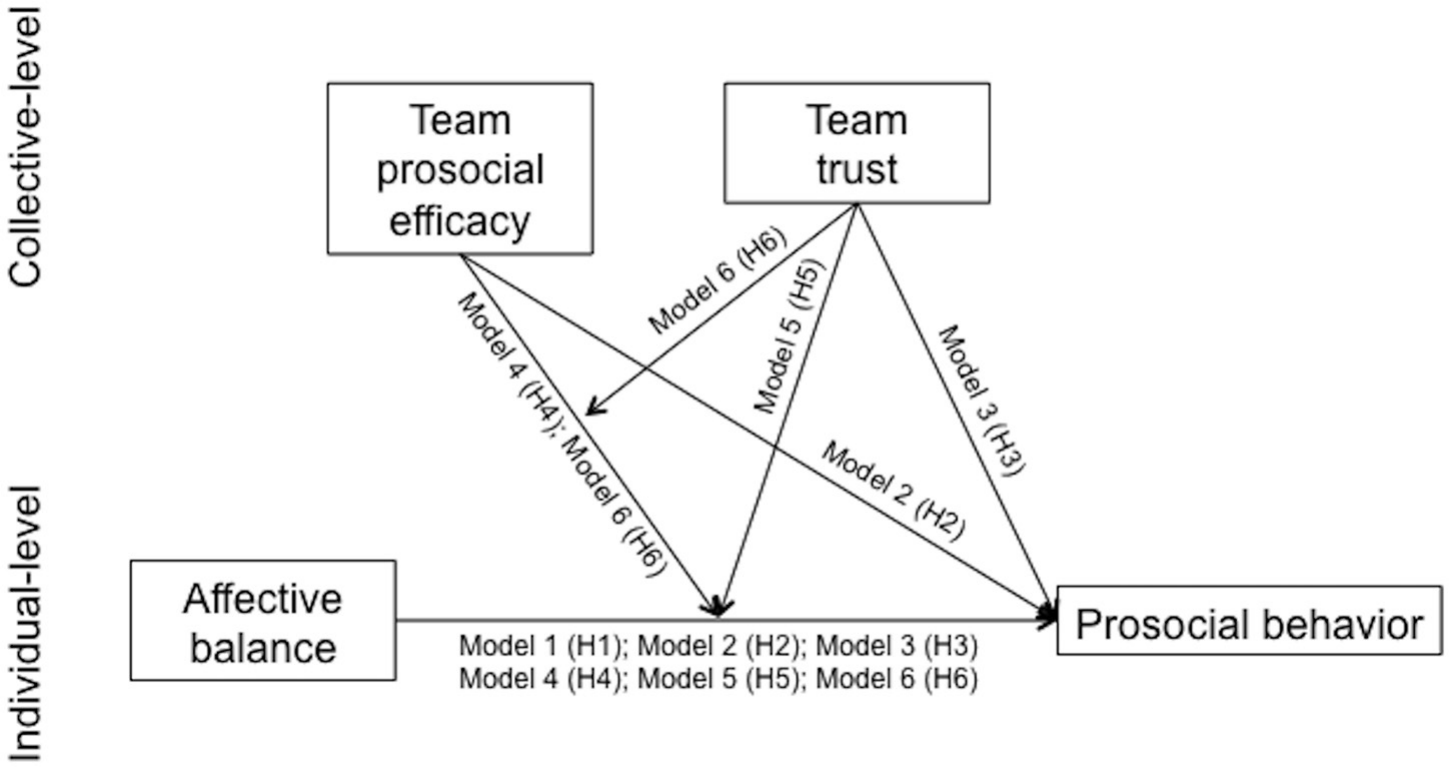
Multilevel model of prosocial behavior. In Model 1, the individual-level variable (affective balance; H1) is inserted as predictor of prosocial behavior. In model 2, the group-level variable (team efficacy; H2) is inserted as a predictor of prosocial behavior. In model 3, the group-level variable (team trust; H3) is inserted as a predictor of prosocial behavior. In model 4 the two-way interaction (team efficacy X affective balance; H4) is considered as a predictor of prosocial behavior. In model 5 the two-way interaction (team trust X affective balance; H5) is considered as a predictor of prosocial behavior. In model 6, the three-way interaction (team trust X team efficacy X affective balance) is considered as a predictor of prosocial behavior.

A multilevel study was conducted to analyze individual (affective balance) and collective (team prosocial efficacy and team trust) factors that could potentially explain individuals’ prosocial behavior towards team members. Fig 1 summarizes the multilevel model and hypotheses of this study.

### Overview of this Study

Previous research on the motivational determinants of prosocial behavior has mainly focused on individual variables, rather than group or team variables. Once, the results of those studies are barely applicable to prosocial behavior in teams. This study examined individual (affective balance) and collective (team prosocial efficacy and team trust) variables that potentially influence prosocial behavior towards team members together, using a multilevel framework. We first investigated the association between prosocial behavior and an individual-level factor (affective balance). Then we analyzed the association between prosocial behavior and two team-level factors (team prosocial efficacy and team trust). Finally, we evaluated the cross-level two-way and three-way interactions between the individual and team factors. The interest of this study lies in the attempt to address a gap in the literature on interactions between individual and collective level variables in the prediction of prosocial behavior within the team. The main objective was to identify, through the development of a multilevel model, some predictive individual and collective level variables of prosocial behavior that help teams to increase prosocial behavior rates in their midst. Moreover, although collective efficacy and trust have been investigated as potential predictors of prosocial behavior, their potential moderation of the association between affective state and prosocial behavior has not, to our knowledge, been studied before. The idea that team trust enhances the effect of team efficacy on the association between affective state and prosocial efficacy was also novel and of potential relevance to the promotion of prosocial behavior within teams.

## Methods

### Participants

The participants were 123 students (56.9% women, age range = [18–38 years], *M* = 20.29, *SD* = 3.51) from a Spanish University nested in 45 teams with two (26.67%) or three (73.33%) members.

### Procedure and Experimental Design

Participants were randomly selected from different classes of the university of Córdoba. Written informed consent was not obtained from the participants because participation was entirely voluntary and data were analyzed anonymously. Participants were informed that they could withdraw from the study at any time without penalty. The study was not reviewed nor approved by any institutional review board (ethics committee) before the study began because the Spanish Ministry of Science and Innovation requires this kind of revision and approval only when the studies imply:

1. Clinical human experimentation
2. Using human embryonic stem cells, or derived therefrom, from pre-embryos remaining lines
3. Use of tissues or biological samples of human origin.
4. Use of personal data, genetic information, etc.
5. Animal Experimentation.
6. Use of biological agents of risk to human health, animal or plant.
7. Use of genetically modified organisms (GMOs). h. Release of GMOs

Participants arrived in groups of approximately 30 and were asked to sit at numbered computers and go a web page to take an online survey and participate in some group tasks.

At the beginning of the procedure data on affective state and several socio-demographic variables (age, sex, educational level, career and leisure activities) were obtained using an online questionnaire created with the Global Park survey program. The questionnaire was referred to as a ‘personal profile questionnaire’ to ensure the reliability of the subsequent false teams generated by the computer, supposedly on the basis of the participants’ personal profiles.

After this participants were informed that the computer had analyzed their responses to the personal profile questionnaire and that they had been incorporated into a team with other participants who exhibited the same personal profile. This procedure was used to generate a sense of team membership. All participants were located in a room with numbered computers and, to find and locate their team partners, the computer number of the other participant(s) appeared on the screen. Then, a manipulation check was performed; we measured participants’ feeling of team membership in order to assess whether our manipulation had the expected effect.

Finally, the team-level variables (team prosocial efficacy and team trust) and then behavioral variable were assessed. Prosocial behavior was assessed using an online group task—the resources dilemma game—that allowed participants and their fellow team members to earn money. At the end of the survey participants were probed for suspicion, fully debriefed and thanked. No identifying information was collected from participants at any point during the procedure.

### Measures

#### Affective balance

An affective balance score was obtained from a short version of the Positive Affect and Negative Affect Scale [39]. Participants responded to 12 items using a seven-point Likert scale (1 = strongly disagree; 7 = strongly agree) to indicate the extent to which each item represented how they felt at that moment. Their affective balance was obtained by subtracting [18] the score for the six negative items (e.g. anger; α = .84) from the score for the six positive items (e.g. happy; α = .84).

#### Team variables

Team prosocial efficacy (α = .93) i.e. the extent to which a participant felt that his or her team was capable of prosocial behavior, was assessed using a five-item scale (e.g. ‘My group can behave cooperatively’) created according to Bandura’s guide for constructing self-efficacy scales [40]. Participants recorded their answers using a seven-point Likert scale (1 = strongly disagree; 7 = strongly agree).

Team trust (α = .71) was assessed using an adaptation of the trust scale used by Greenhalgh and Chapman [41]. The scale included three items (e.g. ‘I feel that those two people can be counted on to help me’) that reflected the confidence participants had in their interactions with the other member(s) with whom they were completing the group tasks. The seven-point Likert scale was presented after the participants were assigned partners for the online tasks.

To support the aggregation method for the team variables, we calculated inter-member reliability (ICC1 and ICC2), we computed the r*_WG(J)_ index of reliability, and we tested whether average scores significantly differed across teams (indicated by an *F* test from a one-way analysis of variance contrasting team means on each variable). ICC1 indicates the proportion of variance in ratings due to team membership, whereas ICC2 indicates the reliability of team mean differences [2, 42]. Good support for aggregation was obtained for team prosocial efficacy [r*_WG(J)_ = 0.77; *F*(44, 122) = 1.89; *p* < .01; ICC1 = 0.25; ICC2 = 0.47] and team trust [r*_WG(J)_ = 0.56; *F*(44, 122) = 1.80; *p* < .01; ICC1 = 0.23; ICC2 = 0.45].

#### Prosocial behavior: sharing with team members

Prosocial behavior (*r* = .67, *p* < .001) was measured using a resources dilemma game. In each round of the game members of the team were free to take as much as they wanted from a monetary fund given to the team as a whole. Participants did not know how much other team members were taking. They were informed that if the total amount of money taken by the team was higher than the total amount in the team’s fund then none of the team members would receive any money and that any money remaining in the fund at the end of the game would be split equally among the team. Two rounds were played.

#### Manipulation check: sense of team membership

We assessed sense of team membership by asking participants to indicate their agreement with three items (‘I like the group in which I am’; ‘I want to remain a member of this group’ and ‘I feel attracted to this group’) using a seven-point Likert scale (1 = strongly disagree; 7 = strongly agree) (α = .92).

### Data Analysis

We used a multilevel approach. A multilevel modeling technique was selected because both the data (participants nested within groups) and the main hypotheses (the impact of team prosocial efficacy and team trust on prosocial behavior towards team members; moderation of the relationship between affective balance and prosocial behavior towards team members by team prosocial efficacy and team trust and the moderation of team-trust in the cross-interaction between affective balance, team prosocial efficacy and prosocial behavior) were multilevel in nature [43]. To examine the impact of the individual and collective variables on prosocial behavior, we estimated a series of multilevel random models using the Hierarchical Linear and Nonlinear Modeling software (version 7.01) [44]. Affective balance was measured at the individual level (Level 1), and efficacy and trust were measured at the collective-level (Level 2). At each step in the model building process, every parameter was inspected individually to assess the significance of the residual variance. Individual and aggregated data are available in S1 and S2 Files respectively.

We first ran an intercept-only model (Model 0) to assess how much of the total variance of prosocial behavior should be ascribed to each level of analysis.

Model 0 equations:

$$\text{Level 1: }DilemmaGame_{ij}=\beta_{0j}\;+\;r_{ij}$$

$$\text{Level 2: }\beta_{0j}=\gamma_{00}+u_{0j}$$

Then three further models were estimated. Model 1 estimated the relationship between the individual-level predictor (affective balance; H1) and prosocial behavior.

Model 1 equations:

$$DilemmaGame_{ij}=\beta_{0j}+\beta_{ij}\times \left(AffectiveBalance_{ij}\right)+r_{ij}$$

$$\begin{array}{l} \text{Level 2: }\beta_{0j}=\gamma_{00}+u_{0j}\\ \beta_{1j}=\gamma_{10}+u_{1j} \end{array}$$

In model 2 we added the estimation of the associations between the collective-level predictor prosocial team-efficacy (H2) and prosocial behavior.

Model 2 equations:

$$\text{Level 1: }DilemmaGame_{ij}=\beta_{0j}+\beta_{ij}\times \left(AffectiveBalance_{ij}\right)+r_{ij}$$

$$\begin{array}{l} \text{Level 2: }\beta_{0j}=\gamma_{00}+\gamma_{01}\times \left(TeamEfficacy_{j}\right)+u_{0j}\\ \beta_{1j}=\gamma_{10}+u_{1j} \end{array}$$

In Model 3 we added the estimation of the association between the group-level predictor team trust (H3) and prosocial behavior.

Model 3 equations:

$$\text{Level 1: }DilemmaGame_{ij}=\beta_{0j}+\beta_{ij}\times \left(AffectiveBalance_{ij}\right)+r_{ij}$$

$$\begin{array}{l} \text{Level 2: }\beta_{0j}=\gamma_{00}+\gamma_{01}\times \left(TeamEfficacy_{j}\right)+\gamma_{02}\times \left(TeamTrust_{j}\right)+u_{0j}\\ \beta_{1j}=\gamma_{10}+u_{1j} \end{array}$$

Two models including individual, collective and cross-level two-way interaction were estimated to test specific hypotheses concerning the moderating effect of collective predictors—team-efficacy (Model 4: H4) and team-trust (Model 5: H5)—on the association between the individual-level predictor (affective balance) and prosocial behavior.

Model 4 equations:

$$\text{Level 1: }DilemmaGame_{ij}=\beta_{0j}+\beta_{ij}\times \left(AffectiveBalance_{ij}\right)+r_{ij}$$

$$\begin{array}{l} \text{Level 2: }\beta_{0j}=\gamma_{00}+\gamma_{01}\times \left(TeamEfficacy_{j}\right)+\gamma_{02}\times \left(TeamTrust_{j}\right)+u_{0j}\\ \beta_{1j}=\gamma_{10}+\gamma_{11}\times \left(TeamEfficacy_{j}\right)+u_{1j} \end{array}$$

Model 5 equations:

$$\text{Level 1:}DilemmaGame_{ij}=\beta_{0j}+\beta_{ij}\times \left(AffectiveBalance_{ij}\right)+r_{ij}$$

$$\begin{array}{l} \text{Level 2: }\beta_{0j}=\gamma_{00}+\gamma_{01}\times \left(TeamEfficacy_{j}\right)+\gamma_{02}\times \left(TeamTrust_{j}\right)+u_{0j}\\ \beta_{1j}=\gamma_{10}+\gamma_{11}\times \left(TeamTrust_{j}\right)+u_{1j} \end{array}$$

Model 6 included individual, collective and cross-level three-way interactions to test the specific hypothesis concerning the conditional effect of all the individual and collective-level predictors on prosocial behavior (H6).

Model 5 equations:

$$\text{Level 1: }DilemmaGame_{ij}=\beta_{0j}+\beta_{ij}\times \left(AffectiveBalance_{ij}\right)+r_{ij}$$

$$\begin{array}{l} \text{Level 2: }\beta_{0j}=\gamma_{00}+\gamma_{01}\times \left(TeamEfficacy_{j}\right)+\gamma_{02}\times \left(TeamTrust_{j}\right)+u_{0j}\\ \beta_{1j}=\gamma_{10}+\gamma_{11}\times \left(TeamEfficacy_{j}\right)+\gamma_{12}\times \left(TeamTrust_{j}\right)+u_{1j} \end{array}$$

All continuous predictors were grand-mean centered to facilitate the interpretation of main and conditional effects [45]. Finally, models were estimated as either fixed or random error terms depending on the statistical significance of results of preliminary analyses to ensure convergence [45].

In order to calculate how much of the variance in prosocial behavior depended upon the team to which individuals belonged, for the intercept-only model (Model 0) we calculated the Intraclass Correlation Coefficient (ICC), which represents the proportion of variance that lies between teams [46]. And in order to calculate the amount of variance accounted for the predictors in each model, we calculated the *Pseudo R* ^*2*^ statistic; this statistic was calculated by comparing the variance component in the unconditional model (Model 0) to the same variance component in each conditional model [46].

## Results

### Manipulation Check

Descriptive analysis showed that participants felt a strong sense of team membership (*M* = 5.95, *SD* = 1.15), indicating that the manipulation was effective.

### Preliminary Analyses

Descriptive statistics and correlations are provided in Table 1. In accordance with H1, at the individual level affective balance was positively correlated with prosocial behavior, and at the collective level team prosocial efficacy was positively correlated with team trust.

**Table 1:** Means, standard deviations and correlations for all the variables.

	*M*	*SD*	1	2
**Individual level (*N* = 81)**				
1. Affective balance	3.52	0.69	α = .84	
2. Prosocial behavior	17.84	8.27	.43***	-
**Collective level (*N* = 30)**				
1. Team prosocial efficacy	6.32	0.69	-	
2. Team trust	5.98	.80	.73***	-

*** *p* < .001.

### Multilevel Analyses

Table 2 summarizes the results of the multilevel hypothesis tests.

**Table 2:** Results of the multilevel regression predicting individual prosocial behaviour.

	Model 0^(1)^	Model 1	Model 2	Model 3	Model 4	Model 5	Model6
**Fixed effects (with robust standard errors)**							
Intercept	17.575***	17.139***	17.203***	17.239***	17.410***	17.397***	17.411***
Affective balance (AB)		2.370*	2.140*	2.137*	2.109*	2.002**	2.044**
Team efficacy (TE)			-1.506(*ns*)	-2.443 (*ns*)	-2.051 (*ns*)	-2.958 (*ns*)	-2.801 (*ns*)
Team trust (TT)				1.040 (*ns*)	1.070 (*ns*)	2.096 (*ns*)	1.976 (*ns*)
AB*TE					3.089*	-	-
AB*TT						3.420*	-
AB*TE*TT							2.969**
**Variance components (Random effects)**							
Within individuals, σ^2^	48.13	27.15	27.72	27.81	28.15	29.07	28.95
Intercept, τ	9.30	9.03	9.13	8.92	8.86	8.05	8.16
χ^2^	65.567*	78.978***	74.639**	71.801**	71.290**	68.363**	68.721**
*d*.*f*.	44	44	43	42	42	42	42
**Deviance**							
(-2*log likelihood)	842.23	803.10	801.31	796.54	791.71	789.03	784.03
Estimated Parameters	2	4	4	4	4	4	4
**Pseudo R2 (% explained compared to Intercept Only Model)**							
	-	43.59%	42.41%	42.22%	41.51%	39.60%	38.85%

*p < .05;

**p < .01;

***p < .001

^(1)^ 26.74% of the variance in prosocial behavior depended on the team to which the individual belonged.

Model 0 demonstrated that there was significant variance between teams for the level 1 outcome variable (χ2 = 66.57; *df* = 44; *p* < .02). Approximately 26.74% of the variance in prosocial behavior scores depended upon the team to which an individual belonged (*ICC* = .2674).

Model 1 indicated that affective balance was a positive predictor of prosocial behavior, confirming H1.

Model 2 demonstrated that team prosocial efficacy did not predict prosocial behavior—thus failing to corroborate H2—but did indicate that affective balance remained a predictor of prosocial behavior in the presence of collective-level control variables—thus providing additional support for H1.

Model 3 demonstrated that team trust did not predict prosocial behavior—failing to corroborate H3—although affective balance remained a predictor of prosocial behavior in the presence of collective-level control variables—providing more support for H1.

**Fig 2:**
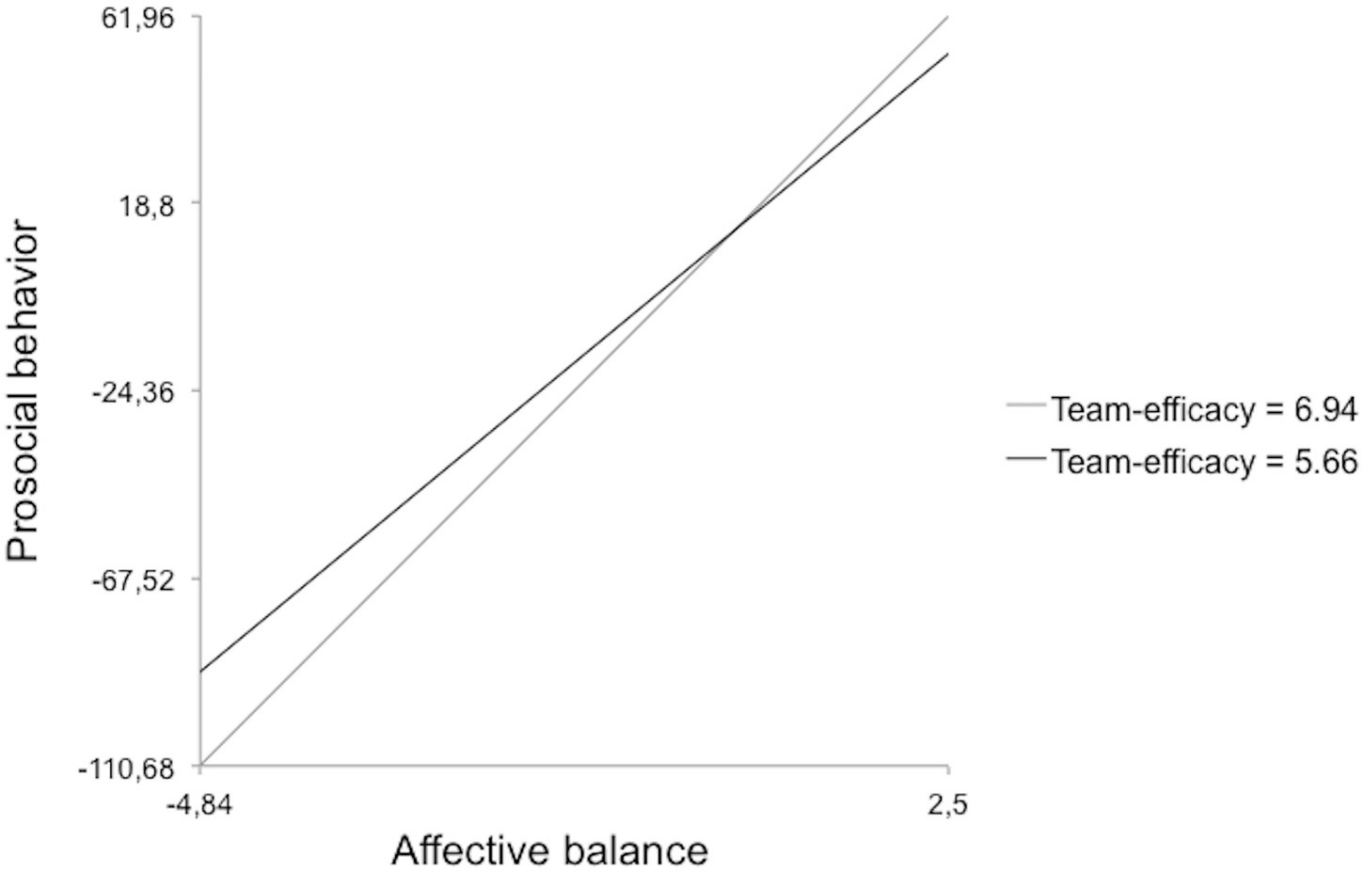
Cross-level two-way interaction effect for team prosocial efficacy.

Model 4 implied the reliability of the two-way cross-level interaction between affective balance, team-efficacy and prosocial behavior—team prosocial efficacy moderated the effect of affective balance on prosocial behavior—confirming H4. Affective balance had a greater effect on prosocial behavior in teams with higher team efficacy (Fig 2).

**Fig 3:**
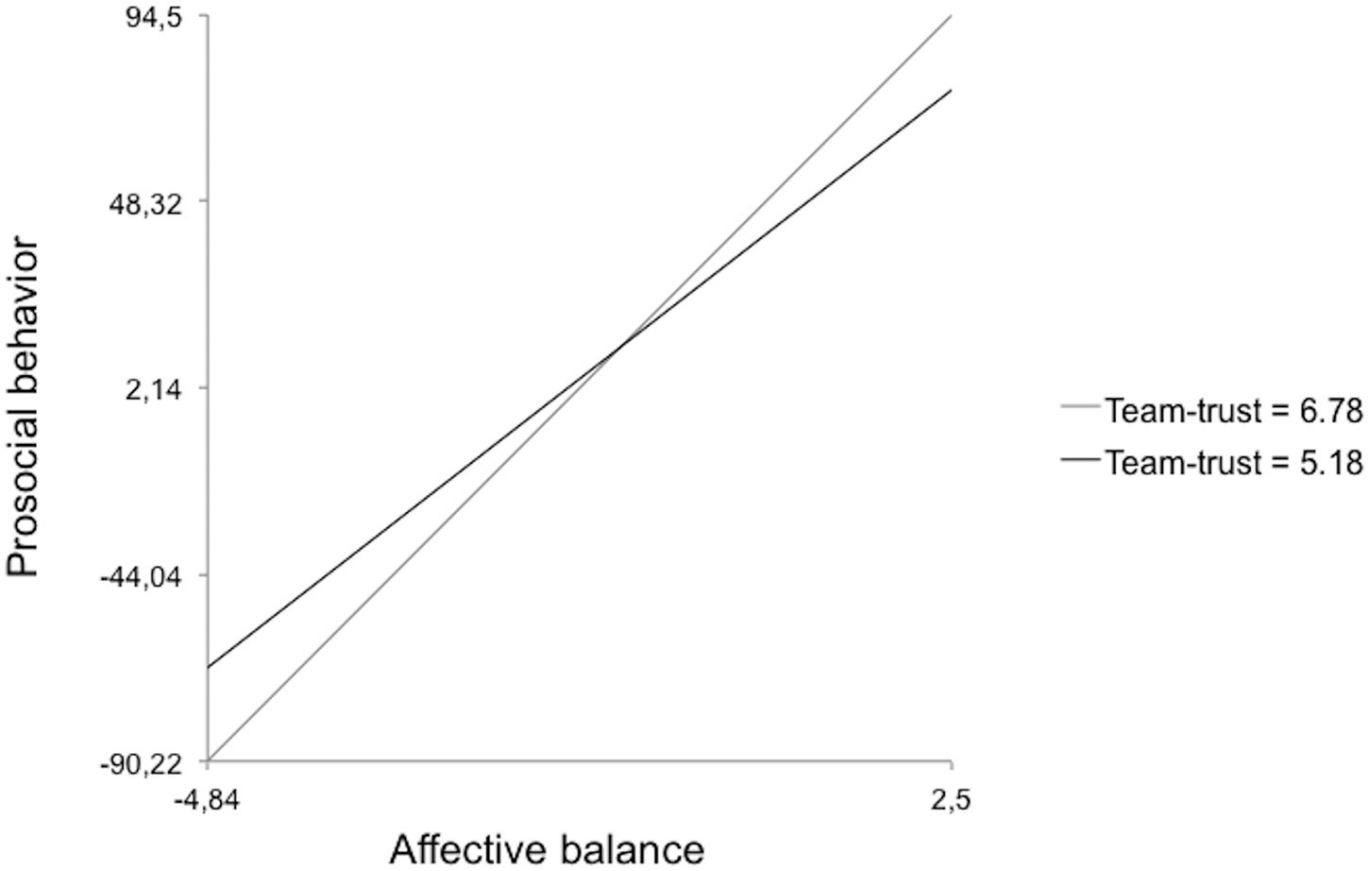
Cross-level two-way interaction effect for team trust.

Model 5 implied the reliability of the two-way cross-level interaction between affective balance, team trust and prosocial behavior: team trust moderated the effect of affective balance on prosocial behavior, thus confirming Hypothesis 5. Affective balance had a greater effect on prosocial behavior in teams with higher trust levels (Fig 3).

**Fig 4:**
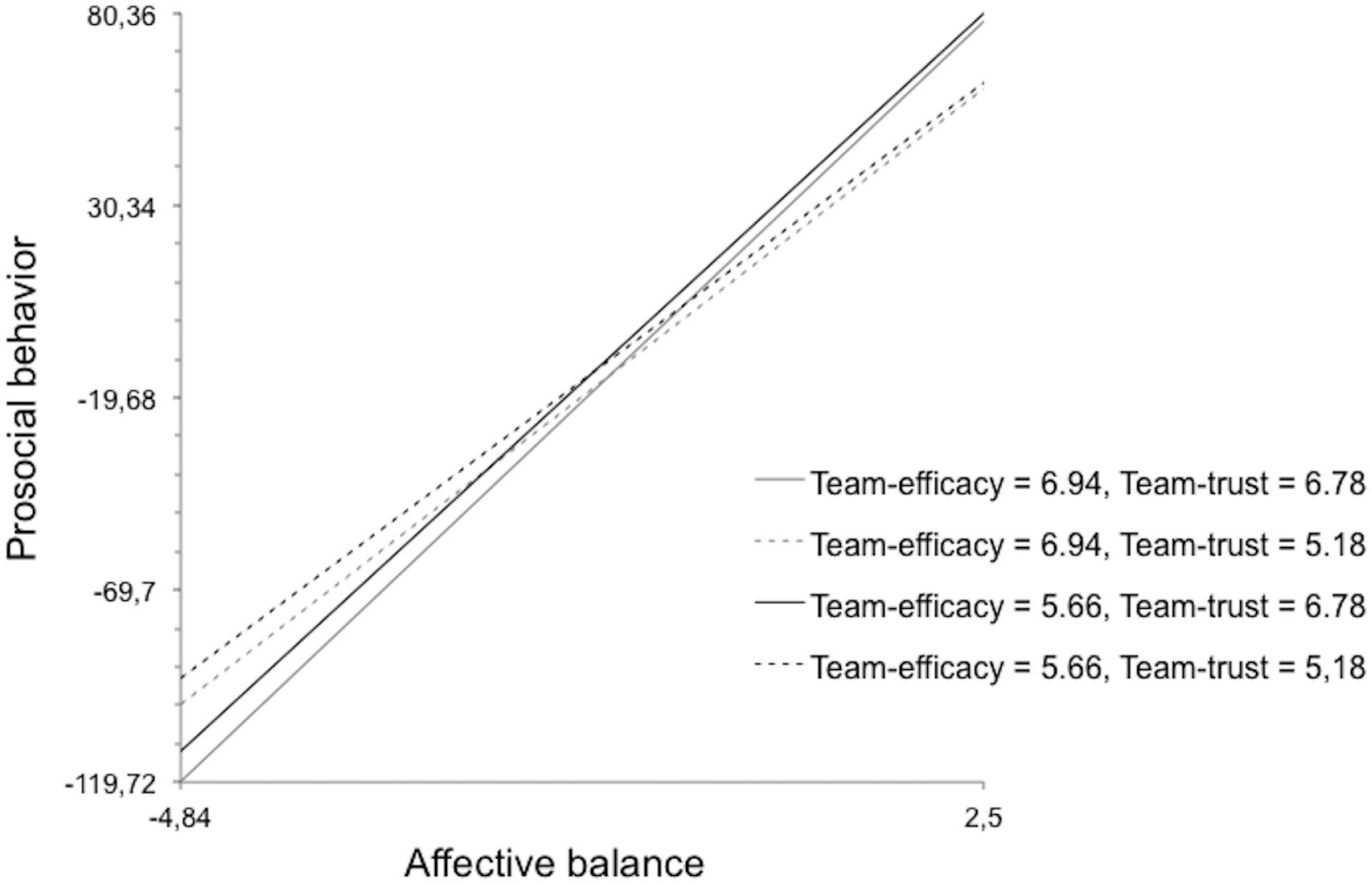
Cross-level three-way interaction effect.

Model 6 revealed a three-way interaction: team trust moderated the previously described cross-level interaction between affective balance, team-efficacy and prosocial behavior; the effect of team prosocial efficacy on the relationship between affective balance and prosocial behavior depended on team trust (Fig 4), thus providing support for H6. The interaction between affective balance, team-efficacy was most pronounced in the context of high team trust.

## Discussion

In this study we used a multilevel framework to examine the combined influence of individual- (affective balance) and team-level (team prosocial efficacy and team trust) factors on the prosocial behavior of individuals towards team members. Taken together, our findings allow us to draw several conclusions. First, we have provided a more detailed account of the multilevel mechanisms by which affective balance—at the individual-level—and team prosocial efficacy and team trust—at the team-level—influence prosocial behavior within teams. Second, our analysis revealed cross-level interactions involving individual- and team-level variables; this suggests that team prosocial efficacy and team trust are potential mediators of the relationship between affective balance and prosocial behavior. We also found that team trust played an important role in prosocial behavior towards fellow team members; it moderated the relationship between affective balance and prosocial behavior and enhanced the positive effect that team-efficacy had on the association between affective balance and prosocial behavior.

Consistent with previous research [3, 4], the individual-level variable affective balance was associated with higher levels of prosocial behavior towards fellow team members. In other words we showed that when individuals are in a globally positive emotional state—i.e. feeling good—they behave more prosocially towards fellow members of a team. Our data thus supported our first hypothesis, that individuals in a globally positive emotional state would be inclined to behave prosocially towards fellow team members. This result was in line with previous studies showing that positive affect promotes prosocial behavior [3, 4], whilst negative affect decreases it [17]. At a practical level, these results suggest that it is important to promote a positive affective balance in the members of teams and small groups. As Fredrickson [47] suggested, “although other routes to enhanced positive affective experiences exist (e.g. through diet, exercise, facial feedback), our habits of mind and action provide perhaps the most powerful leverage points for increasing positive affectivity” (p. 454) and proposed that finding positive meaning, being open, doing good, and being social would improve individuals’ global affective state. Stewart, Craig, MacPherson and Alexander [48] proposed a social support-based intervention to promote positive affect among elderly widowed people that could be adapted for use with groups and teams.

Team prosocial efficacy and team trust were also examined as team-level predictors of prosocial behavior among team members, but our second and third hypotheses were not supported. Members of teams with high team prosocial efficacy or high team trust were not more to behave prosocially towards fellow team members: there was no evidence that either team prosocial efficacy or team trust had a direct effect on prosocial behavior. Nevertheless the two-way interaction involving individual- and team-level variables demonstrated that, in teams with high team prosocial efficacy or high team trust there was a stronger association between positive affect and prosocial behavior. If they were feeling good individuals behaved more prosocially towards fellow team members if they belonged to a team with high team prosocial efficacy or high team trust; Thus our fourth and fifth hypotheses were corroborated. These results are consistent with the trait-based interactionist model [36] and seemed to corroborate the assumption that team efficacy and team trust create a positive atmosphere that can activate and enhance the tendency for those that feel good to behave in a specific and positive way [13]; in this study, prosocially. This study thus highlighted the importance of promoting a globally positive emotional state—at individual-level—and higher team prosocial efficacy and team trust—at the collective-level—in social programs which are intended to promote prosocial behavior among teams.

Finally, our results provided evidence that together team efficacy and team trust predict individual behavior [7], in this case prosocial behavior towards team members. Our findings also corroborated previous research indicating that team trust is a more important predictor of prosocial behavior than team efficacy [7]. In our results, the slope was steeper when team trust was higher, regardless of team efficacy; in contrast, when team trust was lower, the slope was less steep, regardless of the level of team prosocial efficacy. This result corroborated our sixth hypothesis: individuals with a globally positive emotional state who belonged to a team with high team prosocial efficacy levels were more inclined to behave prosocially towards team members if the team also had high team trust. Just as Bandura [7] found that group efficacy was insufficient to promote active individual participation in the political domain—high trust was also necessary; we have provided evidence that team efficacy is insufficient to promote prosocial behavior; high team trust is also needed. As we argued in the introduction, in a team with low team trust the negative atmosphere created by the lack of trust among team members decreased (a) the probability of prosocially behavior and (b) the positive influence of a globally positive emotional state on prosocial behavior. Thus, the importance of the team trust levels in the realm of the team was enhanced regarding to the importance of the team prosocial efficacy levels. These results, like those discussed above, also suggest that interventions to promote prosocial behavior among teams should seek to increase team prosocial efficacy and team trust. On the basis of our findings and other research indicating that team culture—competitive or cooperative—influences behavior at individual level [49] we propose that teams should be provided with some sort of cooperative framework, perhaps via formative and educational program that allows individuals to experience cooperation and its practical benefits. Positive experiences of cooperation and prosocial behavior in the team context should enhance (a) the team’s shared belief in its conjoint ability to act prosocially, i.e. team prosocial efficacy and (b) the team members’ shared confidence in one another i.e. team trust.

### Limitations and Future Research

Certain limitations of the research can be identified. Other variables that may co-vary with prosocial behavior were not assessed. Previous research has shown that individual-level variables such as traits (e.g. empathy or prosociality), values, self-efficacy and social support [8, 26, 50–52] and group-level variables such as team commitment, team cohesion and team support [53, 54] play a role in prosocial behavior. In future research it would be interesting to consider potential covariates in our model. It has been shown that the group size [29] and neighborhood size can affect the emergence and sustainability of cooperation [28, 29, 55, 56]. Social interactions are often modeled as complex networks, and the evolution of cooperation can also be modeled using such networks. When a network (i.e. complex systems that contain many members—abstracted to nodes—and connections—abstracted to edges) is weighted, the weight represents the link between nodes and the property and intensity of the connection [57], and thus may describe the frequency of cooperation between members. It has been shown that cooperation is more likely to develop and persist in medium-sized networks than small or large networks; in small or large networks or groups defection rates are higher [28, 55, 56]. This is because in small networks the pay-off for cooperation does not exceed that for defection by enough to promote the spread of cooperation through the network. If, however, interaction size is moderated between neighbors in the network, thus defectors are highly dispersive, then cooperation will spread through the network. In other words “cooperators among cooperative clusters usually have a higher payoff than defectors. With the size of the neighborhood increasing little by little, the dominant payoff for cooperators within the cluster becomes more visible; hence, cooperation is greatly facilitated” [28] (p. 727). In recent years there has been growing interest in the evolution of cooperation in interdependent or multiplex networks [56], future research should determine whether the findings presented here vary as a function of group or network size.

## Conclusions

This research has contributed to our understanding of the effects of motivational variables on within-group prosocial behavior. Moreover, we addressed a gap in the literature, by demonstrating that individual- and collective-level variables jointly influenced—via interaction—individuals’ prosocial behavior towards fellow team members. Our results showed that team efficacy and team trust exerted cross-level effects on the association between personality (affective balance) and behavior (prosocial behavior) by moderating its interaction. We demonstrated that both team efficacy and team trust interacted with and influenced the association between individual global emotional state and prosocial behavior. Moreover, the relevance of team-trust levels was highlighted regarding the team-efficacy levels: when individuals belonged to low trusting teams, although with high levels of team-efficacy (a) they engaged in lower levels of prosocial behavior and (b) the positive slope between affective balance and trust was lower than when they belonged to high trusting teams.

This study highlights the importance of promoting positive affective balance, team prosocial efficacy and team trust as part of efforts to encourage prosocial behavior within a team. It would therefore be beneficial to develop programs to improve affective balance, team prosocial efficacy and team trust for use in the context of group work.

## Supporting Information

S1 File Individual data. Data for individual level variables.
(SAV)

S2 File Aggregated data. Data for collective level variables.
(SAV)
